# On Mindfulness Training for Promoting Mental Toughness of Female College Students in Endurance Exercise

**DOI:** 10.1155/2021/5596111

**Published:** 2021-08-24

**Authors:** Yi Wang, Jing Tian, Qingxuan Yang

**Affiliations:** ^1^School of Physical Education, Weinan Normal University, Weinan 714099, China; ^2^School of Foreign Languages, Weinan Normal University, Weinan 714099, China; ^3^Department of Physical Education, Chang'an University, Xi'an 710064, China

## Abstract

**Objective:**

The aim of this study was to examine the promoting effects of mindfulness training on female college students' mental toughness in endurance exercise.

**Methods:**

A cluster sampling method was used to select 60 female college students as subjects. Based on the body mass index (BMI), stratified randomization was used to divide them into the mindfulness-training group and the control group. Participants in mindfulness-training group had an 8-week mindfulness training, while participants in control group waited. Before and after training, Five Facet Mindfulness Questionnaire (FFMQ) and Connor–Davidson Resilience Scale (CD-RISC) were used for pretest and posttest, and paired *t*-test and covariance analysis were performed on pretest and posttest between-group data.

**Results:**

(1) Paired *t*-test results showed the posttest scores (26.67 ± 3.56; 20.97 ± 3.66; 126.53 ± 8.59) of the three dimensions of description, nonresponse and FFMQ total score of the mindfulness-training group were higher than the pretest scores (25.53 ± 3.74; 19.23 ± 3.59; 121.43 ± 6.78). Statistical significance was shown in their differences (*t* = −2.25; −2.70; −3.25, *p* < 0.05). However, there was no statistical significance in the pretest and posttest of control group. The covariance analyses showed the posttest scores of the mindfulness-training group in three dimensions of description, nonresponse, and FFMQ were higher than the posttest scores of the control group. Statistical significance was shown in their differences (*F* = 6.55; 6.08; 5.91; *p* < 0.05). (2) Paired *t*-test showed posttest scores (46.50 ± 5.93; 30.40 ± 3.75; 15.00 ± 2.34) were significantly higher than pretest scores (42.60 ± 7.68; 26.50 ± 4.32; 12.87 ± 2.51) in all dimensions of the mental toughness of the mindfulness-training group. Statistical significance was shown in their differences (*t* = −3.135, −4.765, −4.922, *p* < 0.01). However, there was no significant difference in the pretest and posttest scores in all dimensions of the mental toughness of the control group. The covariance analysis showed that the posttest scores of all dimensions of the mental toughness of the mindfulness-training group were higher than those of the control group, and the differences were statistically significant (*F* = 11.133, 12.101, 16.053, all *p* < 0.001). (3) Paired *t*-test showed that the posttest score of the mindfulness-training group on exercise intensity perception immediately after 800-meter endurance run (5.67 ± 2.61) was lower than the pretest score (7.03 ± 1.24) and the difference was statistically significant (*t* = 4.18, *p* < 0.001), while the difference was not statistically significant in the control group. The covariance analysis showed that the posttest score of the mindfulness-training group on exercise intensity perception was lower than that of the control group, and the difference was statistically significant (*F* = 15.81, *p* < 0.001).

**Conclusion:**

Mindfulness training improved the level of female college students' mindfulness and mental toughness in their endurance sports, while reducing the fatigue feeling of female college students in endurance sports.

## 1. Introduction

In recent years, “mindfulness” has been widely used as the third generation of cognitive behavioral therapy, which is a psychotherapy method to improve individual psychological flexibility and reduce empirical avoidance by accepting and changing the current state, such as mindfulness-acceptance-commitment [1]. Mindfulness affects cognition and emotion through psychological and behavioral regulation, including attention, memory, and emotional regulation, so as to reduce the occurrence of psychological disorders such as pain sensation and drug dependence [2], anxiety, and depression [3]. The core of mindfulness is to pay attention to and accept the current physical experience [4, 5]. It can reduce the individual's sense of pressure through emotional regulation (the processes by which emotional responses are modified to accomplish individual goals [6]) to improve subjective well-being. Mindfulness training focuses on breathing and body scanning to guide attention to the current experience and get rid of the influence of disturbing information. Guiding consciousness and attention are always maintained on the task object, and open and nonjudgmental acceptance of physical experience to avoid the influence of negative experience on emotion [7].

Mindfulness training, through psychological control and regulation, wakes up the ability of self-regulation of stress and emotion in concentration and attention. Its core is the self-regulation of attention and the psychological attitude of openness and acceptance, including continuous attention and acceptance of physical experience. It is in sharp contrast with avoidance, avoidance of negative experience, and memory [8]. In recent years, more and more studies have proved that mindfulness training plays a positive role in sports. For example, Feng and Si believe that mindfulness training improved the mindfulness level, attention level, and sports performance level of athletes [9]. Through case analysis and logical induction, Liu and Xu believe that there are many similarities between mindfulness training and traditional psychological counseling and training, which can provide a feasible basis for shooting athletes to carry out mindfulness training [10]. Mindfulness training promotes sports performance and reduces anxiety levels [11]. Compared with the traditional psychological training methods, it can more effectively reduce the negative effects of sports behavior and emotion, improve sports performance, and reduce emotional disorder. In sports [12], mindfulness training can improve the athletes' attention and their acceptance level of physical experience. It can also promote the physical feeling of athletes and reduce the negative emotional reaction of athletes [13]. Besides, mindfulness training can also improve the acceptance of pain stimuli [14]. The existing research mainly focuses on high-level athletes, and there are few reports on the influence and promotion of the general population, especially in endurance sports.

Endurance exercise refers to long-term single repetitive task exercise [15]. The aerobic endurance quality level promoted by it is closely related to cardiopulmonary function and is the basic part of physical fitness and an important factor in evaluating physical health. Lack of endurance quality is also the main factor affecting the physical health of Chinese students. However, under certain intensity, long-term repetitive, single endurance exercise will inevitably produce physiological feelings such as dyspnea, heart rate rise, lactic acid accumulation, muscle pain, and other psychological phenomena such as tension, anxiety, and depressive symptoms. Meanwhile, we should also mobilize and maintain attention to overcome the distraction in the process of exercise for a long time. Therefore, participating in endurance sports requires strong mental preparation to initiate an endurance task and keeping attention to the exercise task all the time. Surely, in addition to motivation, will, warming-up, and other influencing factors, strong mental toughness regulated by emotion and attention is also needed to initiate and maintain endurance sports behavior [16].

Early studies reported [17] that mental toughness in sports is the application of personality toughness to sports, which refers to the psychological ability to cope with the pressure in sports, suppress bad emotions, and finally achieve good results [18]. Mental toughness is not only affected by congenital inheritance but also a dynamic and applied developmental personality factor [19]. However, from the perspective of diversification, this paper studies the relationship between sports environment and mental toughness and integrates the sports situations-related concepts of cognition, self-confidence, concentration, and representation into the concept of sports mental toughness, forming the ecological orientation of sports mental toughness. So, it is possible to shape or develop sports mental toughness through psychological intervention [20]. Therefore, the sports mental toughness understood from the categories of cognition, emotion, and behavior can be cultivated and improved through the acquired intervention and training [21]. There is a significant correlation between mental toughness and emotional regulation and coping behavior in a certain situation. Higher mental toughness is significantly correlated with positive behavioral coping strategies such as psychological representation, sense of effort, thought control, and logical analysis [22]. The core of mindfulness is attention and acceptance of the current physical experience. By adjusting breath, body scanning, and attention regulation, consciousness and attention are always kept on the task object. Regulating cognitive, memory, attention, and emotions can help reduce negative emotions such as emotion disorder and anxiety symptoms, get rid of interference information, and improve attention.

Women are more likely to be affected by their emotions under stress. Emotional disorder, as an intermediary variable, is more likely to affect female behavioral inhibition, and experience avoidance is activated as a negative emotion regulation strategy to reduce the persistence of exercise behavior, thus resulting in posttraumatic stress disorder (PTSD) [23]. Therefore, women's sports behavior in endurance sports is more likely to be affected by mental toughness shown in emotions and attention. However, female college students without systematic professional training are more likely to have negative emotions and behavioral inhibition such as tension, anxiety, depression, and so on. Therefore, mental toughness is very important to the endurance sports behavior of female college students. The paper takes female college students from ordinary undergraduate colleges as the research object, through eight weeks of mindfulness training, to verify the promotion effect of mindfulness training on female college students in endurance sports.

## 2. Research Subjects

The calculation of the sample size is an important part of the research design. The determination of the sample size is based on a priori assumptions of effect size, significance level, and power force. The sample size is calculated by G ^*∗*^ power 3.1. Assuming the effect size of 0.5 at a modest level, the significance level of 0.05 [24], and a power of 0.8 when performing a paired *t*-test of intra-group means, 34 participants are required per group. Assuming the preset effect size of 0.7, the significance level of 0.05, and a power of 0.8 when performing an independent sample *t*-test of intergroup means, the participants needed are still 34 each.

Using cluster sampling method, 67 female college students were selected from two natural classes of the same grade and major in an ordinary undergraduate college in the Central and Western China, with the average age of 19.54 ± 0.82, average height of 162.73 ± 5.36 cm, average weight of 55.47 ± 10.47 kg, and BMI 21.06 ± 4.26. According to the BMI index, 67 female college students were divided into 4 levels. Within each level, participants were divided into mindfulness group and control group by completely stratified randomization. The average age of the participants in the mindfulness group was 19.30 ± 0.77, and that of the control group was 19.76 ± 0.82. (*t* = −2.378, *p*=0.020). The average height of participants in the mindfulness group was 162.06 ± 5.12 cm, and that of the control group was 158.72 ± 28.32 cm (*t* = 0.676, *p*=0.503). The average weight of participants in the mindfulness group was 56.64 ± 11.00 kg, and that of the control group was 54.34 ± 9.95 kg (*t* = 0.896, *p*=0.374). The BMI of the mindfulness group was 21.61 ± 4.42, and that of the control group was 20.52 ± 4.09 (*t* = 1.041, *p*=0.302). There was no significant difference in BMI between the mindfulness group and the control group (*t* = 1.067, *p*=0.291). There was no significant difference in age, height, weight, and BMI between the two groups. All the above can be seen in Table 1.

## 3. Research Methods

**Figure 1:**
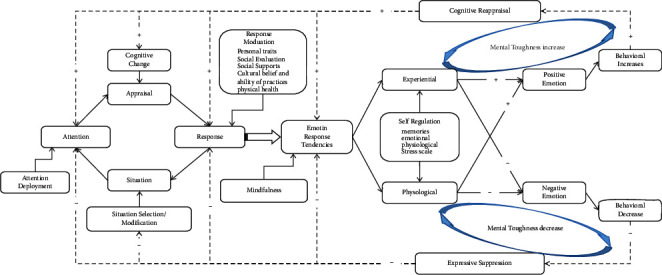
The process model of emotion regulation behavior and mental toughness. Partially quoted from [25]. Dotted line represents emotional regulation after the first round.

Gross established an emotional regulation model in 2015, which believes that situation, attention, evaluation, and response form a closed loop, and their mutual influence forms a spiral upward [25]. This study believes that in addition to the closed loop formed by situation, attention, evaluation, and response, the emotional tendency generated by the response not only has a positive effect on behavior, which spirals upward but also has a negative effect on behavior and makes it spiral downward. The process model of emotion regulation behavior and mental toughness is shown in Figure 1.

The process model of emotional regulation behavior and mental toughness shows that when a situation occurs, individuals choose the situation according to their expectations and then direct their attention to their emotional goals through attention regulation, which determine the individual's evaluation of the situation through their cognitive changes. The response mechanism is then directed to experience, physiology, and behavior through emotion [25]. If both the experience and the physical sensation are positive, they will induce positive emotions, and if both are negative, they induce negative emotions.

When the positive emotion is generated, the behavior in the current status will increase, and the benign behavior experience is produced, which leads to cognitive reappraisal. Cognitive reappraisal can promote positive emotions in situations, attention, assessment, and response. Therefore, cognitive reappraisal can lead to the decline of negative emotional experience levels and the encoding of subsequent memory [26, 27]. When the negative emotion is generated, the behavior in the current status is reduced, and the negative behavior experience is produced, which leads to inhibition of expression. Inhibition of expression can bring about the decrease of positive emotion experience [28] and the increase of response of sympathetic nervous system [29], which will activate the amygdala and other brain regions where emotions are generated [30], resulting in negative memory [31]. Similarly, inhibition of expression will also lead to negative emotional experience in situation, attention, assessment, and response, thus resulting in negative emotional tendency. A virtuous circle formed by cognitive reappraisal will continuously promote the increase of benign behavior, and the behavior will continue, while mental toughness will increase as well. In contrast, the negative cycle formed by inhibition of expression will lead to negative emotions in the situation, attention, evaluation, and response. Thus, the behavior will be reduced or interrupted, resulting in experience avoidance, while mental toughness is reduced too. The method of mindfulness intervention is to regulate the negative cycle of expression inhibition in the situation, attention, evaluation, response, and emotion regulation tendency through mindfulness training so as to reduce the interruption of behavior and improve mental toughness.

### 3.1. Research Tools

#### 3.1.1. The Five-Factor Mindfulness Scale (FFMQ)

It is composed of 39 items in total, 5 dimensions: observation, description, conscious action, no evaluation, and no reaction. The internal consistency coefficient between the scores of the five dimensions and the total score of FFMQ is between 0.792 and 0.905. The five most representative dimensions of mindfulness include observation (attention and attention to various internal experiences and external stimuli), description (description and classification of observed phenomena through words), and conscious action (full involvement at the moment, attention to each experience consciously), no judgment (acceptance of the various experiences at the moment), and no response (no habitual, automatic response in the face of stimulation) [32].

#### 3.1.2. The Psychological Toughness Scale

It is modified on the basis of the original Connor Davidson Resilience Scale (CD-RISC-25), including three dimensions: tenacity (It describes an individual's equanimity, promptness, perseverance, and sense of control when facing situations of hardship and challenges), strength (It focuses on the individual's capacity of recovering and becoming strong after setbacks and past experiences.), and optimism (It reflects the individual's tendency of looking on the positive sides of things and trusting one's personal and social resources. Therefore, this factor is labeled as optimism, measuring one's confidence in resisting adverse events). The Cronbach's *α* coefficient was 0.91 [33]. It has good reliability and validity and is widely used [34].

#### 3.1.3. The Subjective Fatigue Scale

The “Rating of Perceived Exertion” (RPE) was used to monitor participants' internal training load. “Rating of Perceived Exertion” (RPE), which has a single item, was originally used in the field of medicine [35]. RPE is used in the sports and exercise sciences to indicate the psychological feedback of physiological exercise intensity. It is the psychological load effect of exercise intensity and reflects the influence of exercise behavior on cognition, attention, emotion, etc. [36]. Studies have proved that “self-perception of exertion” can be used to indicate and detect the intensity of exercise [35, 37]. The application of RPE in sports and exercise science is recomposed and applied by Foster [38]. There are many ways to divide the scale of RPE. Among them, the 0–10 CR-10, which is called Session-rating of perceived exertion Scale, is regarded as the most widely used. SRPE has been proved to be an effective method for evaluating training load [39–42]. The correlation between SRPE and objective index such as heart rate, blood lactic acid, and VO2max was *r* = 0.62, 0.57, and 0.64, respectively [43]. Liu et al. made appropriate modifications to the English version of the Rating of Perceived Exertion and translated it into Chinese [44]. Chen et al. verified the validity of the Chinese version of SRPE through the testing of football players. It is considered that the correlation between SRPE value after training and training load calculated by heart rate is between 0.75 and 0.91, so SRPE can effectively quantify and evaluate the training load of athletes [45].

### 3.2. Experimental Design

The experiment adopted a mixed experimental design of 2 (experimental group and control group) × 2 (pretest and posttest). Before the experimental intervention, the mindfulness group and the control group were asked to complete the scales of the Five-factor Mindfulness Scale and Psychological Toughness Scale, respectively, and 800-meter endurance running. Besides, all the participants were asked to form the Subjective Fatigue Scale within three minutes after the 800-meter endurance race test. All the work done above is taken as a pretest plan. Then the mindfulness group was given mindfulness intervention for 8 weeks, while the control group participated in class activities normally. After 8 weeks, the two groups were tested with the same scheme as the pretest. In order to eliminate the impact caused by the environment, the posttest was carried out at the same place and environment as the pretest, under conditions similar to the pretest as close as possible in temperature and time.

### 3.3. Mindfulness Training Program

In the study, the Mindfulness-Acceptance-Insight-Commitment (MAIC) training method developed by Si et al. was used [46], as seen in Table 2. Mindfulness training was implemented once a week for 90 minutes in 8 weeks. The course was based on the MAIC training method, with appropriate modifications to increase endurance exercises. The program aimed to train students to develop attention on the body, breathing sensation, sounds, visual objects, thoughts, and emotions. In addition to classroom training, students in the mindfulness group were required to do a daily practice of mindfulness breathing and body description. While in the control group, students were trained in a traditional physical training method by the same coach of mindfulness group in the same field and the same periods of the day, such as gymnastics shoulder elbow stand exercises, gymnastic swan balance training, swallow balance, stretching exercises, strength, and running exercises, etc.

### 3.4. Data Collection Methods

Before and after the intervention, the teacher organized the students into groups to finish the Five-Factor Mindfulness Scale (FFMQ), the Mental Resilience Scale (CD-RISC-25), the 800-Meter Endurance Run, and the Subjective Fatigue Scale, respectively. All the scales were distributed, filled in, and collected in person. The test results were input into SPSS software by a double-input method.

### 3.5. Statistical Processing

SPSS22 software was used for data sorting and statistics, and the measurement data were expressed by mean ± standard deviation. *T*-test was used for general data, paired sample *t*-test was used for intra-group comparison, and analysis of covariance was used for posttest intergroup comparison. Differences were considered statistically significant when *p* < 0.05.

## 4. Results and Analyses

### 4.1. Comparison of Mindfulness Levels

Before the intervention, there were no statistically significant differences between the mindfulness group and the control group in the pretest scores of mindfulness observation (*t* = 0.218, *p*=0.828), description (*t* = 0.368, *p*=0.714), conscious action (*t* = −0.852, *P* = 0.398), no evaluation (*t* = 0.690, *p*=0.49382), and no reaction (*t* = 0.691, *p*=0.492). Paired *t*-tests were performed on the pretest and posttest scores of the mindfulness group and the control group, respectively. It was found that the difference between the pretest and posttest scores of the control group was not statistically significant (*t* = 0.160, −1.444, 0.368, 0.157, −1.346, *p* > 0.05), while the posttest scores in the three dimensions of “description,” “no reaction,” and FFMQ total scores of mindfulness group were higher than those in pretest, and the difference between the pretest and posttest scores of the mindfulness group is statistically significant (*t* = −2.246, −2.695, −3.25, all *p* < 0.05). In the research, the posttest scores of five dimensions of mindfulness were taken as dependent variables, pretest scores as covariates, and the groups as fixed factors for the covariance analysis; it was found that the posttest scores of “observation,” “no response,” and FFMQ total score in mindfulness group were higher than those in the control group, and the differences between groups were statistically significant (*t* = 6.55, 6.080, 5.91, all *p* < 0.05). See Table 3 for an overview of paired *t*-test and covariance analyses of pre- and posttest scores of the mindfulness group and control group.

### 4.2. Comparison of Mental Toughness

Before the intervention, there were no statistically significant differences between the mindfulness group and control group in the pretest scores of mental toughness in three dimensions of tenacity (*t* = 0.172, *p*=0.864), strength (*t* = −0.936, *p*=0.353), and optimism (*t* = −1.263, *p*=0.212). Paired *t*-tests were performed on the pretest and posttest scores of the mindfulness group and the control group, respectively, the result of which showed that the posttest scores of the mindfulness group in three dimensions of “tenacity,” “strength,” and “optimism” (46.50 ± 5.93), (30.40 ± 3.75), (15.00 ± 2.34) were higher than its pretest scores, respectively (42.60 ± 7.68), (26.50 ± 4.32), (12.87 ± 2.51), and the differences between the pretest and posttest scores of the mindfulness group were statistically significant (*t* = −3.135, −4.765, −4.922, *p* < 0.01) while the differences between the pretest and posttest scores of the control group were not statistically significant. In the research, the posttest scores of the mindfulness group in three dimensions were taken as the dependent variable, the pretest scores as the covariates, and the groups as the fixed factors, respectively; for the covariance analysis, it was found that the posttest scores of mindfulness group in three dimensions were higher than those in the control group, and the differences between groups were statistically significant (*t* = 11.133, 12.101, 16.053, all *p* < 0.001). See Table 4 for an overview of paired *t*-test and covariance analysis of the pre- and posttest scores of mental toughness in the mindfulness group and the control group.

**Figure 2:**
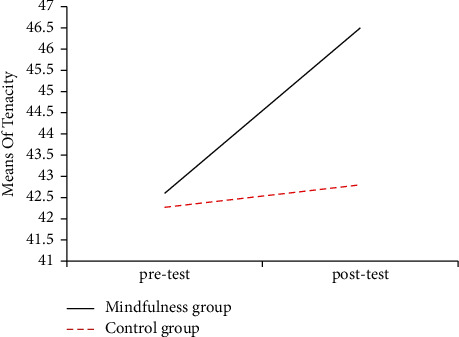
The mean scores of the tenacity of pre-and posttest in the mindfulness group and the control group.

**Figure 3:**
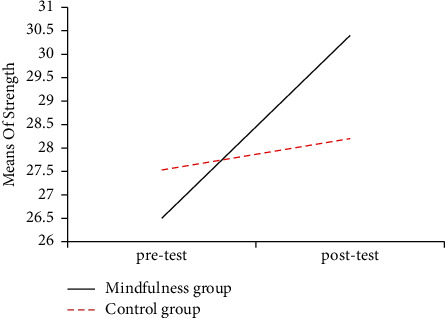
The mean scores of the strength of pre-and posttest in the mindfulness group and the control group.

**Figure 4:**
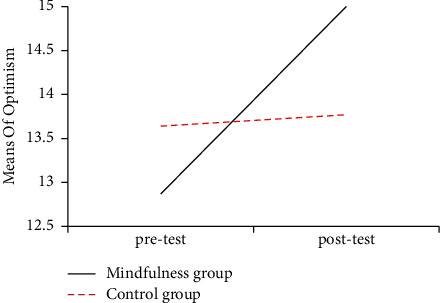
The mean scores of optimistic of pre-and posttest in the mindfulness group and the control group.

The figures of mean scores of mindfulness group and control group in three dimensions of mental toughness showed intuitively that the posttest scores of the mindfulness group in three dimensions were significantly improved compared with its pretest scores and its posttest scores becoming much higher than the posttest score of control group too. Mean changes in the three dimensions of mental toughness are displayed in Figures 2–4 .

### 4.3. Comparison of Exercise Intensity Perception

Paired *t*-test was conducted on the pretest and posttest scores of the exercise intensity perception immediately after two 800-meter endurance runs in the mindfulness group and control group. Paired *t*-test showed that the posttest score of the mindfulness-training group on exercise intensity perception immediately after 800-meter endurance run (5.67 ± 2.61) was lower than the pretest score (7.03 ± 1.24) and the difference was statistically significant (*t* = 4.18, *p* < 0.001), while the difference was not statistically significant in the control group.

The results showed that compared with the pretest score, the posttest score of the mindfulness group on the exercise intensity perception decreased significantly, and the difference was statistically significant (*p* < 0.001), while the control group slightly increased but not statistically significant. In the research, the posttest scores of exercise intensity perception as dependent variables, the pretest scores as covariates, and groups as fixed factors, respectively, for the covariance analysis, it was found that the posttest scores of the mindfulness group on exercise intensity perception were lower than those of control group, and the difference was statistically significant (*p* < 0.001), which can be seen in Table 5.

**Figure 5:**
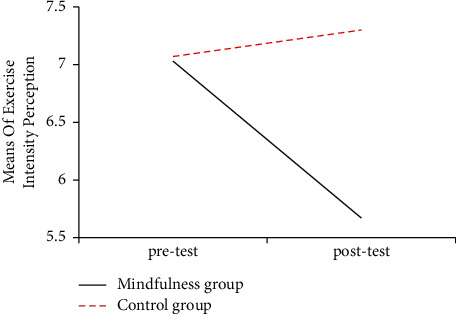
Diagram of the average scores of exercise intensity perception before and after the mindfulness group and the control group.

The means of exercise intensity perception showed that after the mindfulness training intervention, the fatigue of the 800-meter endurance running test in the mindfulness group decreased significantly compared with the control group and before the intervention. Mean changes of exercise intensity perception are displayed in Figure 5.

## 5. Discussion

In 2016, Abdul thought that mindfulness plays an important mediating role in the relationship between the mental toughness and athletic performance of college track and field athletes [47]. Walker's research on women hockey players in 2016 showed that mindfulness is not only significantly related to the overall mental toughness but also closely related to confidence, constancy, and control. Athletes with higher mindfulness levels reported higher control, constancy, and general mental toughness. It was believed the higher the mindful level, the higher the mental toughness level of the athletes [48]. Many previous studies have proved that mindfulness plays an important mediating role in the mental toughness of track and field athletes or long-distance runners, but no empirical research has been conducted. In this study, cluster sampling, stratified randomized block controlled experimental design was adopted, through 8 weeks mindfulness training experimental intervention, to verify the effect of mindfulness training on female college students' mental toughness in endurance sports. The results showed that the female college students who had received the mindfulness training had a significant improvement in the overall level of mindfulness and the scores of some dimensions. In the 800-meter endurance run test, they also showed better mental toughness than the performance before the intervention training and the performance of the control group as well; at the same time, mindfulness training also reduced college female students' exercise intensity perception and other negative physical feelings such as fatigue. Through empirical study, this research proves that mindfulness training has a promoting effect on college female students' mental toughness in endurance sports. The results further support Petrillo's research that mindfulness training, as a kind of psychological training, intervenes and improves mindfulness, sports anxiety-related worries, and long-distance runners' expectations [49]. Thompson believes that mindfulness training significantly improves the mindfulness and endurance performance of long-distance runners [50]. Nien believes that mindfulness training can improve college athletes' mindfulness level, endurance performance, and various cognitive functions, including executive functions [51].

In recent years, some scholars have proposed that cognition, self-confidence, concentration, representation, behavioral coping, and other factors related to sports situations should be included in the research field of mental toughness from a diversified perspective, forming a new concept and category of mental toughness. They believe that cognition, emotion regulation, and behavioral coping strategies play important roles in mental toughness [20]. The core of mindfulness is an open and nonjudgmental attention and acceptance attitude towards the current physical experience. Mindfulness training is a new generation of cognitive behavioral therapy for mental disorders, which regulates cognition, memory, and emotion by adjusting attention and acceptance attitude [52].

Mindfulness training is to guide consciousness and attention on the task object through adjusting breathing, body scanning, and attention regulation. Through the regulation of cognition, memory, attention and emotion, it can reduce the influence of exercise-related worries and exercise-irrelevant thoughts (two aspects of cognitive interference during sports) on sports. Besides, mindfulness training trained the participants to accept their physical experience openly and nonjudgmentally, avoid the influence of negative experiences on their emotions, reduce the exercise-related physical worries, and improve the self-cognition related to attention regulation and arousal regulation [53]. Mindfulness training uses breathing, body scanning, experiencing drinking and eating, walking, balance, running, and other body awareness control and adjustments to guide attention to stay focused on the task experience at the current moment so as to get rid of the influence of irrelevant attention and interference information on the process of endurance exercise, reduce the occupation of working memory capacity by irrelevant attention and interference information during long-term endurance exercise, and improve the working memory ability and the individual's concentration of endurance exercise tasks. At the same time, the regulation of consciousness and attention through mindfulness improves the individual's inhibition and control over previous negative experiences, reduces the negative impact of female college students' past negative experience of endurance sports on current endurance sports behavior, and improves the individual's executive function [54–56]. Through the open and nonjudgmental experience and attention on thoughts, emotions, and body feelings, new behavioral experience feelings are formed, thereby avoiding experience avoidance caused by previous negative experiences and memories. Therefore, mindfulness training can inhibit negative thoughts, emotions, behaviors, and the competition and interference of negative physiological reactions through conscious adjustment and prevent the influence of subconscious, automatic, and habitual thinking on endurance sports behavior so as to promote the individual's conscious perception of current behavior [57–59], which makes female college students inhabit negative coping strategies such as experience avoidance in the endurance running test, reduces the difficulty of starting the endurance exercise task, and prevents the endurance exercise task from being interrupted. This promotes mental toughness in endurance sports.

The individual's various negative physiological experiences and memories in the previous endurance exercises will increase the individual's painful feelings for the body. It can cause individuals to experience negative emotions such as tension, worries, depression when facing a current similar situation and lead to experience avoidance, even stress disorder, which is an important factor affecting female college students to participate in endurance sports. Therefore, Jones believes that individual Pain Catastrophizing mediates the relationship between mindfulness and endurance sports performance, which is an important factor affecting female college students' participation in endurance sports [60]. However, in addition to the physiological reactions of the body itself (accelerated heartbeat, dyspnea, and muscle pain) and the release of chemicals that cause pain during endurance exercise, the individual's perception of pain is also affected by the individual's judgment, expectations, and other emotions and cognition. Individual psychological differences, such as memory, coping strategies, and personality, can inhibit or enhance individual's perception of noxious activities [61]. Meta-cognitive beliefs about worry play an important role in the connection between pain behavior and pain catastrophizing. Positive cognitive beliefs about worry mediate the relationship between neuroticism and pain catastrophizing, while negative cognitive beliefs about worry mediate the relationship between pain catastrophizing and self-reported pain behavior [62]. Pain is variable, and an individual's emotion and cognition are components of pain perception. Therefore, the openness and nonjudgmental attitude towards physical experience is used in mindfulness exercises to cultivate and improve the individual's acceptance and tolerance of pain and other negative physical experiences [63, 64] so as to reduce the negative impact of negative experience on the current endurance sports task and the avoidance coping strategies, which has been proved in many studies in recent years [65–67]. Therefore, mindfulness training improves the individual's open and nonjudgmental acceptance of physical experience, which makes the individual accept, accommodate, and not reject kinds of pain in endurance sports, and improves the tolerance of pain in endurance sports, avoid negative coping such as experience avoidance in endurance sports, so as to improve the mental toughness in endurance sports.

Martin's survey of the sample of cyclists in 2018 found that mindfulness partially mediated a negative relationship between mental toughness and pain catastrophizing. Mindfulness is positively associated with mental toughness and negatively associated with pain catastrophizing [68]. This empirical study proves the theories and hypotheses above and proves that mindfulness training can reduce female college students' negative physical feelings of intensity, pain, and fatigue in endurance sports and improve their mental toughness in endurance sports.

## 6. Limitations and Future Perspectives

It is important to note that although reliable statistical findings have been observed, there are still some limitations in this study. The questionnaire was handed out and collected face to face, and all the subjects were required to fill in the questionnaire carefully, but it is not sure that all participants have given honest answers. In the process of the experimental intervention, a total of 90 minutes of training was conducted once a week for a total of 8 weeks in mindfulness training group. Besides, participants in the mindfulness group were asked to do 45 minutes of mindfulness training on their own every day, while the control group received a certain amount of traditional training every day. However, the individual's intensity of self-training cannot be controlled, so there are some differences, which may affect the results of the experiment. The research data relies on the individual's self-report rather than the observation of the individual's behavior (such as the degree of fatigue during exercise). So the social desirability of the individuals may affect the experimental findings to some degree. Furthermore, the sample size of the study is relatively small. Although it basically meets the requirements of the study, if the sample size is appropriately expanded, a more convincing effect size will be obtained. In future research, we will use more scientific experimental design and control to compare the effects of long-term and short-term mindfulness training on mental toughness in endurance sports. How different intensity, duration, and practice environments in mindfulness training affect individual's mental toughness will also be studied later.

## 7. Conclusion

The level of mental toughness in endurance exercise is the key factor that affects the participation and persistence of endurance sports, while cognition, attention, and emotion before and during the sports are all the factors affecting the persistence of endurance sports behavior. At the same time, endurance exercise may cause anxiety symptoms for low sports or sedentary people. Stubbs et al. conducted a cross-sectional study based on community data in 47 countries around the world and found that there was a significant positive correlation between low physical activity and anxiety symptoms [69].

The emotional states in these sports behaviors are the factors that cause experience avoidance behaviors and new emotional disorders such as anxiety and depression. Traditional psychological training develops internal state and self-control through cognitive behavior methods and techniques such as goal-setting, arousal-control, self-efficacy, and self-talk. However, more and more works of literature believe that trying to suppress negative internal experiences and thoughts will activate them on the contrary, which is the paradox effect of suppression [70]. Mindfulness-Acceptance-Commitment (MAC) proposes acceptance of the present experience, rather than changing, inhibiting, or controlling, which emphasizes attention and nonjudgement. Therefore, mindfulness training promotes the level of mindfulness of female college students through attention guidance and regulation by guiding breathing, body scanning, body movement, etc. The attention adjustment guidance awareness and attention are always maintained on the task object. Mindfulness training promotes the mindfulness level of female college students through attention guidance and regulation, including guiding breathing, body scanning, body movement, etc. Attention regulation guides awareness and attention on the task object consistently. Besides, through the regulation of cognition, memory, attention, and emotions, mindfulness training reduces exercise-related physical worries and the occupation of working memory capacity by some exercise-irrelevant thoughts so as to improve female college students' working memory ability and executive ability of endurance exercise tasks. Through the open and nonjudgmental experience and attention to the endurance sports task, female college students can have a new experience and avoid the experience avoidance caused by the previous negative experience. Therefore, mindfulness can improve the individual's tolerance to pain and other negative body feelings, avoid the negative coping behaviors such as experience avoidance in endurance sports, reduce the female college students' negative body feelings such as intensity feeling and pain, reduce the fatigue feeling of endurance sports, and improve the female college students' psychological resilience in endurance sports. Mindfulness improves the individual's tolerance for pain and other negative body sensations, avoids negative coping behaviors such as experience avoidance in endurance exercise, reduces the female college students' negative physical sensations such as intensity feeling pain and fatigue in endurance exercise, and improves their mental toughness in endurance sports at last.

## Figures and Tables

**Table 1:** Baseline characteristics of the mindfulness and control groups.

	Mindfulness intervention group (*n* = 33)		Control group (*n* = 34)		*T*-test	
	Mean	Std.	Mean	Std.	*T*	Sig. (2-tailed)
Age (years)	19.30	0.77	19.76	0.82	−2.378	0.020
Height (cm)	162.06	5.12	158.72	28.32	0.676	0.503
Weight (kg)	56.64	11.00	54.34	9.95	0.896	0.374
BMI (kg/m^2^)	21.61	4.42	20.52	4.09	1.041	0.302

Note. BMI: body mass index.

**Table 2:** Contents of mindfulness training.

Week	Training subjects	Training contents
Week 1	Approaching mindfulness and preparation for mindfulness training	1. Introduce mindfulness to the participants. 2. Do a brief centering exercise
Week 2	Understanding mindfulness in practice	1. Practice mindful breathing 2. Do a body scan from head to toes.
Week 3	Attention regulation	1. Practice mindful breathing 2. Do 30 minutes of sitting meditation. 3. Listen carefully to possible sounds with mindfulness
Week 4	Acceptance	1. Practice mindfulness eating with raisins. 2. Taste water, an apple, or a banana with 7 steps: hold, look, touch, smell, release, swallow, and feel.
Week 5	Value and awareness	1. Gymnastic shoulder and elbow stand exercises, 2. Mindfulness stretching exercises.
Week 6	Commitment	1. Gymnastic swan balance training 2. Mindfulness walking
Week 7	Skills practice	1. Endurance training of double arms horizontal lift. 2. Mindfulness running exercises.
Week 8	Integrating training	Do systematically integrating mindfulness training

**Table 3:** Paired *t*-test and covariance analyses of pre- and posttest scores of mindfulness group and control group (Mean ± SD.).

	Paired *t*-test							*p*-value	ANCOVA		
	Mindfulness intervention group (*n* = 33)				Control group (*n* = 34)				*F*	*Pp*-value	Partial *η*^2^
	Pretest	Posttest	*t*	*p*-value	Pretest	Posttest	*t*				
Observation	24.70 ± 6.05	26.13 ± 4.37	−1.973	0.058	24.40 ± 4.49	24.32 ± 3.18	0.160	0.874	6.55	0.013	0.103
Description	25.53 ± 3.74	26.67 ± 3.56	−2.246	0.032	25.20 ± 2.25	25.83 ± 2.36	−1.444	0.160	1.376	0.246	0.024
Conscious action	27.80 ± 5.76	28.50 ± 3.89	−0.789	0.436	28.87 ± 3.72	28.68 ± 2.72	0.368	0.716	0.133	0.717	0.002
No evaluation	24.17 ± 3.47	24.27 ± 4.56	−0.135	0.893	24.80 ± 3.63	24.72 ± 2.92	0.157	0.876	0.008	0.928	≤0.001
No reaction	19.23 ± 3.59	20.97 ± 3.66	−2.695	0.012	18.63 ± 3.11	19.15 ± 1.80	−1.346	0.189	6.080	0.017	0.096
FFMQ total score	121.43 ± 6.78	126.53 ± 8.59	−3.25	0.003	121.90 ± 6.71	122.70 ± 5.50	−0.77	0.45	5.91	0.018	0.094

**Table 4:** Paired *t*-test and covariance analysis of the pre- and posttest scores of Mental Toughness in the mindfulness group and the control group (Mean ± SD.).

	Mindfulness intervention group (*n* = 33)			*p*	Control group (*n* = 34)			*p*-value	ANCOVA		
	Pre-test	Post-test	*T*		Pre-test	Post-test	*T*		*F*	*p*	Partial *η*^2^
Tenacity	42.60 ± 7.68	46.50 ± 5.93	−3.135	0.004	42.27 ± 7.35	42.80 ± 6.02	−1.262	0.217	11.133	0.001	0.163
Strength	26.50 ± 4.32	30.40 ± 3.75	−4.765	≤0.001	27.53 ± 4.22	28.20 ± 3.71	−1.455	0.156	12.101	0.001	0.175
Optimism	12.87 ± 2.51	15.00 ± 2.34	−4.922	≤0.001	13.63 ± 2.17	13.77 ± 2.22	−0.724	0.475	16.053	≤0.001	0.220

Tenacity: Describing an individual's equanimity, promptness, perseverance, and sense of control when facing situations of hardship and challenge. Strength: Focusing on the individual's capacity of recovering and becoming strong after set back and past experiences. Optimism: Reflecting the individual's tendency of looking on the positive sides of things and trusting one's personal and social resources, measuring one's confidence in resisting adverse events.

**Table 5:** Paired *t*-test and covariance analysis of the pre- and posttest scores of exercise intensity perception in the mindfulness group and the control group (Mean ± SD).

	Mindfulness intervention group (*n* = 33)			*p*	Control group (*n* = 34)			*p*	ANCOVA		
	Pre-test	Post-test	*T*		Pre-test	Post-test	*T*		*F*	*p*	Partial *η*^2^
Exercise intensity perception	7.03 ± 1.24	5.67 ± 2.61	4.18	≤0.001	7.07 ± 1.57	7.30 ± 1.49	−1.02	0.315	15.81	≤0.001	0.217

## Data Availability

The data are available upon request.
